# Cooling Reduces cAMP-Stimulated Exocytosis and Adiponectin Secretion at a Ca^2+^-Dependent Step in 3T3-L1 Adipocytes

**DOI:** 10.1371/journal.pone.0119530

**Published:** 2015-03-20

**Authors:** Mickaël F. El Hachmane, Ali M. Komai, Charlotta S. Olofsson

**Affiliations:** Department of Physiology/Metabolic Physiology, Institute of Neuroscience and Physiology, The Sahlgrenska Academy at University of Gothenburg, Gothenburg, Sweden; Tohoku University, JAPAN

## Abstract

We investigated the effects of temperature on white adipocyte exocytosis (measured as increase in membrane capacitance) and short-term adiponectin secretion with the aim to elucidate mechanisms important in regulation of white adipocyte stimulus-secretion coupling. Exocytosis stimulated by cAMP (included in the pipette solution together with 3 mM ATP) in the absence of Ca^2+^ (10 mM intracellular EGTA) was equal at all investigated temperatures (23°C, 27°C, 32°C and 37°C). However, the augmentation of exocytosis induced by an elevation of the free cytosolic [Ca^2+^] to ~1.5 μM (9 mM Ca^2+^ + 10 mM EGTA) was potent at 32°C or 37°C but less distinct at 27°C and abolished at 23°C. Adiponectin secretion stimulated by 30 min incubations with the membrane permeable cAMP analogue 8-Br-cAMP (1 mM) or a combination of 10 μM forskolin and 200 μM IBMX was unaffected by a reduction of temperature from 32°C to 23°C. At 32°C, cAMP-stimulated secretion was 2-fold amplified by inclusion of the Ca^2+^ ionophore ionomycin (1μM), an effect that was not observed at 23°C. We suggest that cooling affects adipocyte exocytosis/adiponectin secretion at a Ca^2+^-dependent step, likely involving ATP-dependent processes, important for augmentation of cAMP-stimulated adiponectin release.

## Introduction

White adipose tissue is an endocrine organ that secretes a variety of hormone-like protein factors, commonly referred to as a*dipokines*. Adiponectin is a protein hormone secreted exclusively from white adipose cells and involved in modulation of glucose and lipid metabolism [1].

Regulated exocytosis has been investigated in several neuroendocrine cell types and is known to involve Ca^2+^, ATP and temperature-dependent steps [2]. Adiponectin has been suggested to be secreted via regulated exocytosis of a pre-stored pool of secretory vesicles [3,4] but very little is known about stimulus-secretion coupling in the white adipocyte. Our recent work has shown that adipocyte exocytosis/adiponectin secretion is stimulated via PKA-independent cAMP-stimulation in a Ca^2+^-independent manner and is amply augmented by a combination of Ca^2+^ and ATP [5]. Thus, unlike archetypal endocrine cell types, Ca^2+^ amplifies but does not trigger exocytosis in adipocytes.

Characterisation of the steps of stimulus-secretion coupling, such as vesicle replenishment and fusion [2], is crucial in order to understand exocytosis. Several studies have shown that Ca^2+^-dependent exocytosis exhibits temperature-dependent components in both neuronal [6] and endocrine [7–9] cell types. Temperature-dependent exocytotic mechanisms were first described in catecholamine secreting chromaffin cells and the temperature-sensitive step was suggested to represent a rate-limiting late exocytotic component [7]. A similar temperature-dependent enhancement of exocytosis was reported in pancreatic beta-cells [8] and melanotrophs [9] and was suggested to reflect an increased supply of vesicles ready for release. Moreover, insulin-stimulated translocation of glucose transporter 4 (Glut4) containing vesicles in white adipocytes is inhibited by temperature reduction [10].

Here we have investigated the temperature dependence of exocytosis and short-term adiponectin release in 3T3-L1 adipocytes, a widely accepted and commonly used model of primary adipocytes. The aim of the study was to elucidate mechanisms important in regulation of white adipocyte stimulus-secretion coupling. Electrophysiological recordings of exocytosis (increase in membrane capacitance) were compared to measurements of secreted adiponectin under similar conditions. We report that a temperature drop is without effect on cAMP-stimulated exocytosis/adiponectin secretion but that cooling completely abolishes the augmentation of exocytosis/secretion induced by Ca^2+^.

## Material and Methods

### Cell culture and differentiation

**Fig 1:**
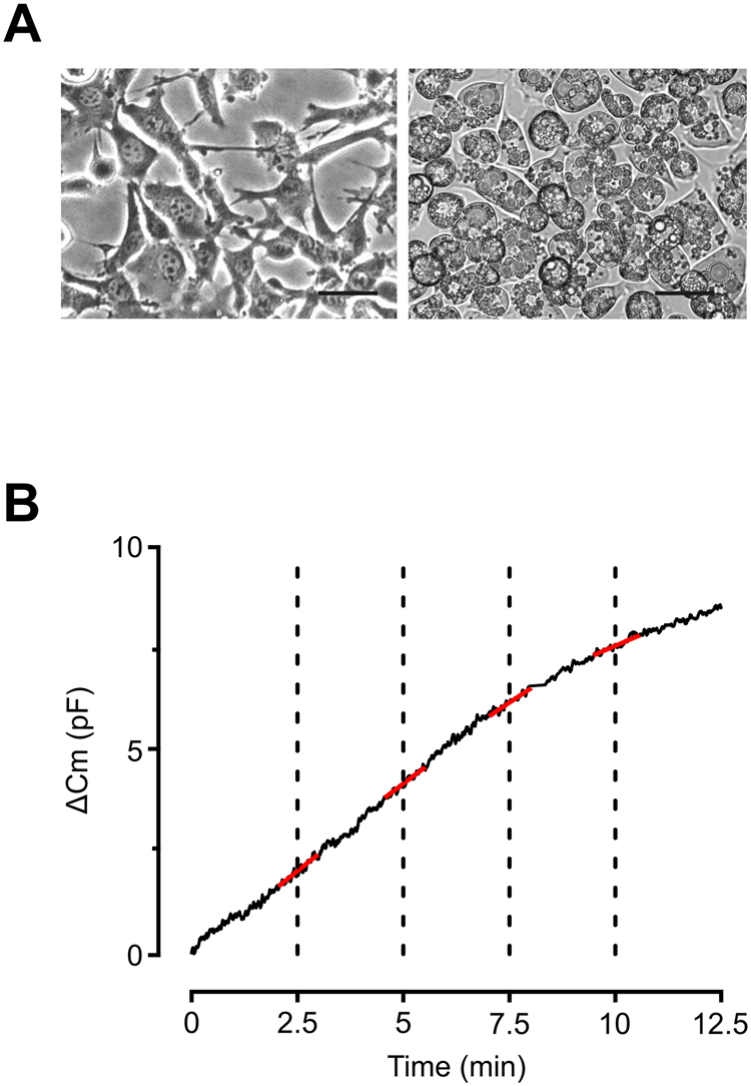
Undifferentiated and mature 3T3-L1 adipocytes as well as demonstration of analysis performance. **A** Example of 3T3-L1 adipocytes in the fibroblast-like state before differentiation (left) as well as when differentiated into mature adipocytes (right). **B** Representative capacitance recording with Δ*C*_m_ (delta membrane capacitance) plotted against time showing how analyses were carried out. Δ*C*/Δ*t* was measured at the time intervals indicated by the dotted lines by fitting straight functions to the data points (red lines superimposed on the black capacitance trace). Scale bar = 50μm.

3T3-L1 adipocytes (mouse origin) were maintained and differentiated according to established protocols [11] and as previously described [5]. Experimental studies were carried out in differentiated 3T3-L1 adipocytes between day 7 and 9 from start of differentiation. The level of differentiation was visually determined (degree of lipid filling) and only cells regarded to be fully mature (as determined by the intracellular presence of large lipid droplets) were chosen for experiments (Fig. 1A). In adiponectin secretion studies, only wells with a visually assessed differentiation level of ≥95% were used.

### Electrophysiology

Exocytosis was measured as increase in membrane capacitance [12] using the whole-cell configuration of the patch-clamp technique and an EPC-9 patch-clamp amplifier (HEKA Electronics, Lambrecht/Pfalz, Germany and PatchMaster software). Patch pipettes were pulled from borosilicate glass capillaries and coated with Sylgard (Dow Corning Corporation). Pipettes were heat-polished prior to use. The pipette resistance was typically 3-5 MΩ when filled with pipette-filling solution and access resistance after establishment of the whole-cell mode was ≤10 MΩ. Cells were voltage-clamped at -70 mV during the recordings.

3T3-L1 adipocytes, grown in Petri culture dishes (Nunc, Denmark), were continuously superfused with an extracellular solution (EC) containing (in mM) 140 NaCl, 3.6 KCl, 2 NaHCO_3_, 0.5 NaH_2_PO_4_, 0.5 MgSO_4_, 5 HEPES (pH 7.4 with NaOH), 2.6 CaCl_2_ and 5 mM glucose (EC). The pipette filling solutions consisted of (in mM) 125 potassium glutamate, 10 KCl, 10 NaCl, 1 MgCl_2_, 10 EGTA, 3 Mg-ATP, 0.1 cAMP and 5 HEPES (pH 7.15 with KOH). The solution was in some experimental series supplemented with 9 mM CaCl_2_ (yielding a free Ca^2+^ concentration of ~1.5 μM calculated using http://www.stanford.edu/~cpatton/maxc.html.). Experiments were conducted at 23°C, 27°C, 32°C or 37°C as specified. The temperature in the cell culture dish was regulated by an in-house feed-back controller.

### Measurements of intracellular Ca^2+^ concentrations

The intracellular Ca^2+^ concentration ([Ca^2+^]_i_) was recorded with dual-wavelength ratio imaging as previously described [13]. Briefly, cells were cultured in glass bottom dishes (MatTek Corporation, USA) and loaded with the membrane-permeable acetoxymethyl ester of fura-2 (fura-2 AM; Life Technologies) during 1 hour in EC at room temperature with gentle shaking. A Lambda DG-4 illumination system was employed (Sutter Instrument Company, USA) and images were captured using a QuantEM 512SC CCD camera (Photometrics, USA) and MetaFluor software (Meta imaging series 7.5, Molecular Devices, USA). Cells were alternately excited at 340 and 380 nm and emitted light was collected at 510 nm. Ratios were converted into [Ca^2+^]_i_ using equation (5) of [14] and a K_d_ of 224 nM. Values of maximum (R_max_) and minimum (R_min_) ratios as well as S_f2_/S_b2_ were determined using solutions with a high Ca^2+^ concentration (12 mM CaCl_2_ + 10 mM EGTA) or lacking Ca^2+^ (10 mM EGTA alone). Measurements were carried out at 23°C or 32°C as indicated.

### Studies of secreted adiponectin

3T3-L1 adipocytes were grown and differentiated in 6- or 12-well plates (Sarstedt or Nunc, respectively) and secretion studies were carried out as previously described [5]. Briefly, cells were pre-incubated for 30 minutes in EC lacking glucose and in the presence or absence of BAPTA-AM (Life Technologies) as specified. EC, supplemented with test substances as indicated, was added and cells were incubated on gentle shaking for 30 min at 23°C or 32°C in parallel. At the end of incubation the EC was removed, centrifuged, aliquoted and samples were stored at -80°C awaiting analysis. Secreted adiponectin was measured using a mouse Adiponectin ELISA kit (R&D Systems Europe, UK) and expressed relative to total cellular protein (measured with Pierce BCA protein, Thermo Scientific, USA).

### Data analysis

The rate of capacitance increase (Δ*C*/Δ*t*) was measured by applying a linear fit to the curve at specified time points as shown in Fig. 1B. The statistical significance of variance between two means was calculated with one-way ANOVA and Student’s t-test, paired or unpaired as appropriate using OriginPro (OriginLab Corporation, USA). All data are presented as mean values ± S.E.M. for the designated number of experiments.

## Results

### The effect of temperature on 3T3-L1 adipocyte exocytosis

**Fig 2:**
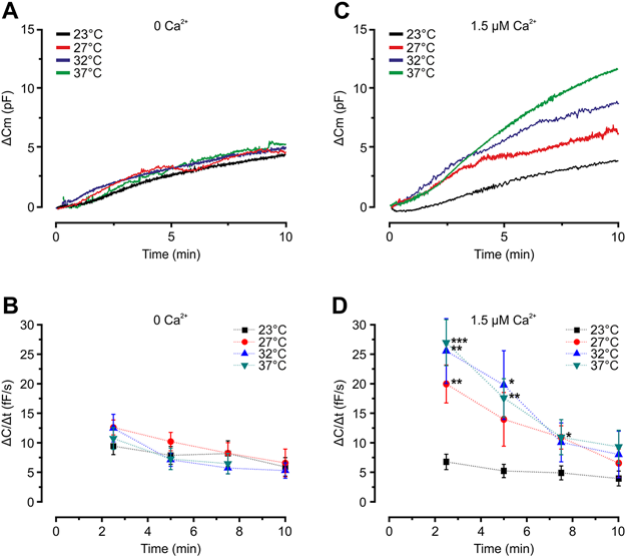
cAMP-stimulated exocytosis is unaffected by cooling while Ca^2+^ potentiation is diminished by temperature reduction. **A** Representative traces of membrane capacitance increases (Δ*C*_m_) evoked by infusion of 0.1 mM cAMP in the absence of intracellular Ca^2+^. **B** Average Δ*C*/Δ*t* at indicated time points using the Ca^2+^ free intracellular solution. Note that Δ*C*/Δ*t* is equal at all times, regardless of the temperature in the cell dish. **C, D** As in (A) and (B) respectively, but with 1.5 μM free Ca^2+^ added to the pipette solution. Observe the Ca^2+^-dependent augmentation of exocytosis as well as the temperature-dependence in the presence of Ca^2+^. n = 7–9; ***P<0.001; **P<0.01; *P<0.05 vs. 23°C. (P = 0.07 for 27°C vs. 23°C at t = 5 min. P = 0.1 at t = 10 for 37°C vs. 23°C)

In order to investigate the temperature dependence of exocytosis in adipocytes, we compared the exocytotic rate at 23°C, 27°C, 32°C and 37°C in cells dialysed with a Ca^2+^-free pipette solution containing 0.1 mM cAMP (10 mM EGTA and 3 mM ATP included) known to trigger adipocyte exocytosis [5]. The rate of exocytosis (Δ*C*/Δ*t*) was measured at the indicated time intervals as shown in Fig. 1B. Exocytosis triggered by intracellular cAMP proceeded at a rate comparable to that reported previously [5] and was not affected by varying the bath temperature (Fig. 2A and B). We next investigated effects of temperature on exocytosis in cells infused with the same solution supplemented with 9 mM CaCl_2_ (free [Ca^2+^] ~1.5 μM). In agreement with what we have previously reported [5], exocytosis was potentiated by inclusion of Ca^2+^ and the rate at 32°C was ≥2-fold amplified compared to 0 Ca^2+^ at all investigated time points (Fig. 2C and D). As shown in Fig. 2C and D, exocytosis was reduced by cooling and the rate at 2.5 min (Δ*C*/Δ*t*_*2*.*5 min*_) after establishment of the whole-cell configuration amounted to only ~30% of that recorded at 32°C or 37°C.

### Q10 values of exocytosis

**Fig 3:**
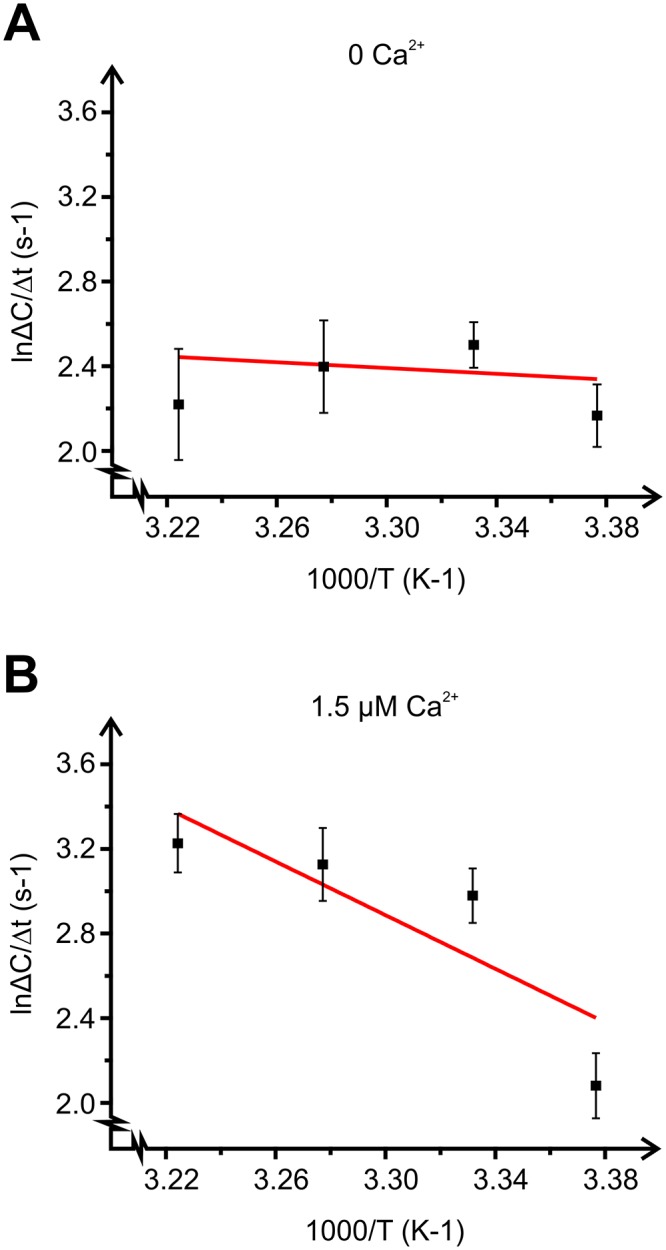
Arrhenius plots presenting the effect of Ca^2+^ on the rate of cAMP-triggered exocytosis. Absolute values of Δ*C*/Δ*t* at t = 2 min plotted against the inverse of temperature (T) in the absence **(A)** and presence **(B)** of cytosolic Ca^2+^. The curve represents a linear least-squares fit to the equation lnk = lnA—E_A_/RT. E_A_ is the energy of activation, k is the change in Δ*C*/Δ*t* upon a temperature change, A is the pre-exponential factor. R represents the gas constant and T the absolute temperature as usual. The negative slope of the curve gives activation energies of 5.7 kJ mol^-1^ (A) and 53 kJ mol^-1^ (B).

The temperature coefficient (Q_10_) can be calculated for the rate of a process as a consequence of raising the temperature by 10°C. While diffusion of ions through their respective channels are modestly affected by a lowering of temperature, biological processes involving enzymatic reactions or protein conformational changes usually display greater temperature dependence (Q_10_ value ≥2) [15]. We estimated Q_10_ values of 1.4 and 3.3 in the absence and presence of 1.5 μM free Ca^2+^ respectively. To further explore the temperature-dependence of adipocyte exocytosis, we constructed Arrhenius plots of exocytotic rates at the temperatures investigated (Fig. 3A and B). The energy of activation (E_A_; slope of fitted line) amounted to 5.7 kJ mol^-1^ in the absence of Ca^2+^ and 53 kJ mol^-1^ in the presence of Ca^2+^. The Q_10_ and E_A_ values indicate that exocytosis in the absence of Ca^2+^ is independent of temperature and requires little energy, whereas Ca^2+^-dependent exocytosis is a much more temperature-dependent and energy demanding process.

### Effects of cooling on 3T3-L1 adipocyte adiponectin secretion

**Fig 4:**
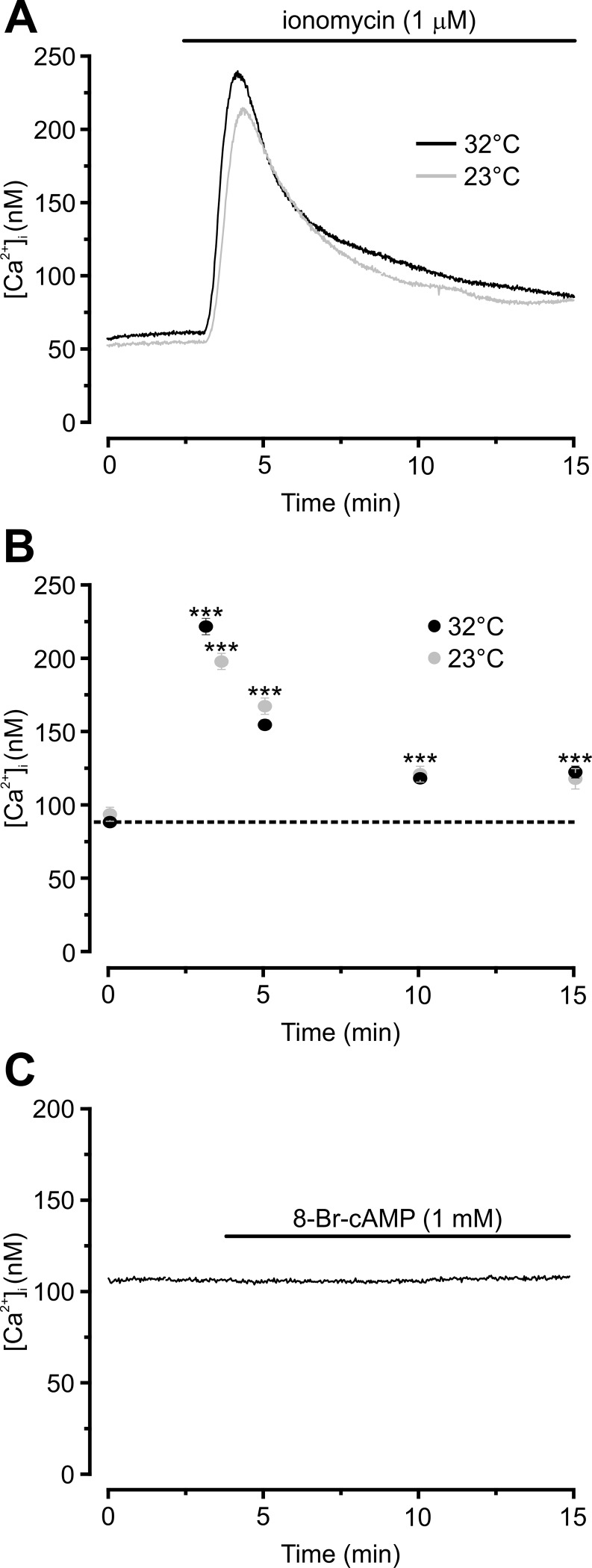
Ionomycin, but not 8-Br-cAMP, elevates adipocyte [Ca^2+^]_i_. Example traces of [Ca^2+^]_i_ responses upon extracellular application of 1 μM ionomycin **(A)** or 1 mM 8-Br-cAMP **(C)**. **B** Average responses to ionomycin at indicated time points between 0 and 15 min. Ionomycin or 8-Br-cAMP was added extracellularly to the dish of cells and remained present throughout the recording as indicated. Note that the peak response to ionomycin shown in (B) was slightly shifted at 23°C (peak at 3.6 min; 5 separate experiments and 122 cells) compared to 32°C (peak at 3.1 min; 4 experiments and 107 cells). The trace in (C) is representative for 101 analysed cells in 4 separate experiments.

3T3-L1 adipocytes secrete substantial quantities of adiponectin [3,16] and we have previously documented a strong correlation between membrane capacitance increases and secretion of this adipokine [5]. In order to compare effects of temperature on exocytosis and adiponectin release, 3T3-L1 adipocytes were incubated in the presence of the membrane permeable cAMP agonist 8-Br-cAMP alone or in combination with the Ca^2+^-ionophore ionomycin (to maximally elevate [Ca^2+^]_i_). Incubations were carried out for 30 minutes at 23°C or 32°C. Effects of ionomycin and 8-Br-cAMP on intracellular Ca^2+^ levels were validated by use of Ca^2+^ imaging. As shown in Fig. 4A, external application of ionomycin (1 μM at 23°C or 32°C) elevated [Ca^2+^]_i_ resulting in a peak at t = ~3 min, after which [Ca^2+^]_i_ declined towards baseline before stabilising at a plateau at 10 min (Fig. 4B). The magnitudes of the responses were comparable although the peak value was slightly (20 nM) but significantly higher at 32°C (P<0.001). However, the measured minute difference is likely of negligible physiological importance. An ionomycin-induced peak [Ca^2+^]_i_ of ~200 nM may appear too small to affect exocytosis but it has to be kept in mind that the measurements show the average [Ca^2+^]_i_ in the cytoplasm and may be several-fold higher at the release sites [17]. Inclusion of 8-Br-cAMP (1 mM) was without effect on [Ca^2+^]_i_ (Fig. 4C; [Ca^2+^]_i_ averaged 108±2 nM before and 119±3 nM after agonist application).

**Fig 5:**
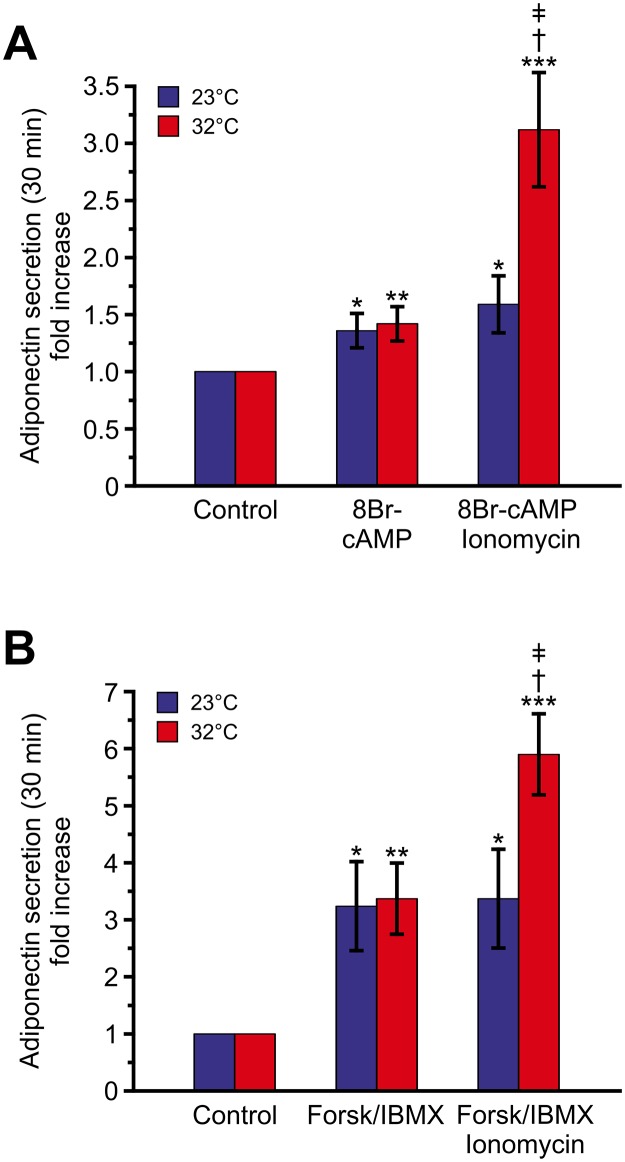
Ca^2+^-augmented adiponectin secretion is abolished by cooling while secretion stimulated by cAMP alone is unaffected. **A** Adiponectin secretion as fold-increase compared to control stimulated by 8-Br-cAMP (1 mM) alone or in combination with ionomycin (1 μM) during 30 min incubations at 23°C (blue) or 32°C (red). **B** As in **(A)** but using forskolin (10 μM) and IBMX (200 μM; forsk/IBMX) as a stimulator. Data are mean values ± S.E.M. of 11 experiments at each temperature in (A) and 7 (23°C) and 8 (32°C) in (B). *P<0.05; **P<0.*01*; **P<0.*001* vs. control at corresponding temperature. †*P<0*.*01* vs. 8-Br-cAMP or forsk/IBMX alone at 32°C; ǂ *P<0*.*05* vs. 8-Br-cAMP + ionomycin or forsk/IBMX + ionomycin at room temperature. The data in (B) at 32°C are the same as in Fig. 7B in [5].

As can be seen in Fig. 5A, 8-Br-cAMP stimulated adiponectin secretion ~1.5-fold over control regardless of the temperature. Ionomycin was unable to augment release further at 23°C, whilst robustly potentiating secretion at 32°C. We further investigated the effect of temperature on adiponectin secretion stimulated by 10 μM forskolin in combination with 200 μM IBMX (forsk/IBMX). We have previously shown that forsk/IBMX elevates 3T3-L1 intracellular cAMP-levels >6-fold [5]. As shown in Fig. 5B, adiponectin secretion was stimulated >3-fold under those conditions, equally at 23°C and 32°C. Inclusion of ionomycin (1 μM) potentiated forsk/IBMX-stimulated secretion ~2-fold at 32°C but was without effect on release stimulated at 23°C. Our secretion data confirm the existence of a temperature-sensitive Ca^2+^-dependent step involved in 3T3-L1 adipocyte stimulus-secretion coupling and further underscore the strong association between 3T3-L1 adipocyte membrane capacitance increases and release of adiponectin.

## Discussion

In this study we have combined electrophysiological recordings of exocytosis with biochemical measurements of adiponectin release in order to elucidate mechanisms involved in regulation of white adipocyte stimulus-secretion coupling. Exocytosis is in archetypal neuroendocrine cells temperature sensitive [6–8]. Our study shows that exocytosis/adiponectin secretion in white adipocytes, in addition to temperature-independent processes, comprises temperature-dependent components involving Ca^2+^. We have recently shown that an elevation of cytosolic Ca^2+^ is necessary in order to achieve robust cAMP-stimulated adiponectin secretion as well as to maintain secretion over longer time-periods (recruitment of new releasable vesicles; [5]). Thus, the temperature-dependence of white adipocyte exocytosis described in this study elucidates controlling mechanisms involved in vesicle recruitment needed for augmentation and long-term maintenance of adiponectin release.

**Fig 6:**
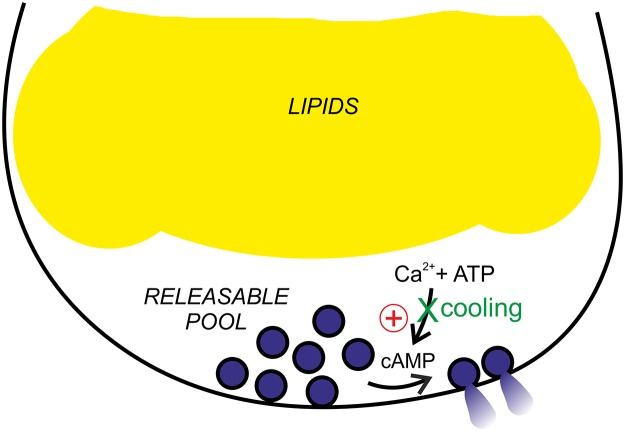
Proposed model of cooling effects on white adipocyte exocytosis. White adipocytes release adiponectin containing vesicles belonging to two functionally distinct populations. cAMP stimulates release of vesicles residing in a readily releasable pool in a temperature-independent manner. The Ca^2+^-dependent augmentation of secretion is reduced/abolished by cooling. Intracellular ATP is necessary for the Ca^2+^ effect. See text for more details.

Our findings are summarised schematically in Fig. 6. We propose that vesicles ready for release may still undergo exocytosis in response to an elevation of cAMP even after cooling, whereas a drop in temperature abrogates more upstream events that are reliant on Ca^2+^. ATP-dependent processes are involved in maintenance of exocytosis in a magnitude of secreting cell types (reviewed in [2]) and this concept can be extended to adipocytes. We have shown that cAMP-triggered release of a readily releasable vesicle pool is unaffected by ATP-deprivation, whereas the Ca^2+^-dependent augmentation of secretion requires the intracellular presence of MgATP [5]. It is conceivable that the effects of cooling presented here in part reflects the necessity of ATP-hydrolysis in order to provide energy for recruitment of new releasable vesicles as well as for phosphorylation of proteins involved in control of exocytosis [2]. However, the rate of enzymatic reactions usually decreases by ~7% for every degree the temperature drops. Thus, the Q_10_ >3 indicate the existence of additional temperature-dependent processes [15].

Our observations in adipocytes are analogous to findings in pancreatic beta-cells [8] and chromaffin cells [7] where secretion of release competent vesicles are unaffected by temperature but vesicle recruitment is reduced by cooling. Also in pituitary melanotrophs, cooling is without effect on a first exocytotic burst corresponding to a pool of releasable vesicles while an ensuing slow phase show great temperature sensitivity [9]. It is interesting that the early and late steps of exocytosis are similarly (un)affected by temperature in adipocytes and other endocrine cell types considering that the pathway of stimulus-secretion coupling differs.

The finding that Ca^2+^ acts as a regulator and not a trigger of adipocyte exocytosis is similar to how mast cell release of inflammatory mediators is controlled. An elevation of cytoplasmic Ca^2+^ is insufficient to trigger exocytosis in this cell type but Ca^2+^ can potentiate secretion triggered by other stimuli, such as the non-hydrolysable GTP analogue GTPgammaS [18]. The shown participation of Epac in white adipocyte exocytosis [5] suggests an involvement of GTP-dependent exocytotic steps. It is thus an exciting possibility that white adipocyte exocytosis, in analogy to what has been shown in mast cells [18] and in some neuroendocrine cell types [19,20], is triggered by GTPgammaS. Interestingly, the magnitude of small G-protein activation has been shown to depend on Ca^2+^ (reviewed in [21]) and hydrolysis of ATP has been suggested to be essential for robust GTPgammaS induced stimulation of exocytosis [19,20].

Our calculated Q_10_ value of 3.3 and the energy of activation (53 kJ mol^-1^) derived from the capacitance recordings are lower than that reported in pancreatic beta-cells (5 and 145 kJ mol^-1^ respectively) [8]. However, insulin secretion is known to be a highly temperature sensitive process, in part due to the rather small size of the readily releasable vesicle pool [8]. Subcutaneous adipose tissue has been suggested to be more important than visceral depots for circulating levels of adiponectin [22,23]. In a physiological context, it seems rational that secretion in subcutaneous adipocytes is less sensitive to cooling since it is situated just beneath the dermis and may accordingly be exposed to low ambient temperatures.

Taken together, the results of this study show that white adipocyte exocytosis/adiponectin secretion encompasses energy-dependent processes. It is conceivable that control of vesicle recruitment signifies an essential mechanism by which the white adipocyte adjusts secretion of adiponectin in response to increased physiological requirements. The fact that adiponectin levels are reduced in obese individuals combined with observations that elevated levels provide protection towards development of type-2 diabetes in normal-weight individuals [24] emphasises the importance of understanding mechanisms controlling release of this adipokine.
